# The Impact of Programmed Cell Death on the Formation of Tertiary Lymphoid Structures

**DOI:** 10.3389/fimmu.2021.696311

**Published:** 2021-07-15

**Authors:** Mélanie Dieudé, Imane Kaci, Marie-Josée Hébert

**Affiliations:** ^1^ Research Centre, Centre Hospitalier de l’Université de Montréal (CRCHUM), Montréal, QC, Canada; ^2^ Department of Microbiology, Infectiology and Immunology, Faculty of Medicine, Université de Montréal, Montréal, QC, Canada; ^3^ Canadian Donation and Transplantation Research Program, Edmonton, AB, Canada; ^4^ Molecular Biology Programs, Faculty of Medicine, Université de Montréal, Montréal, QC, Canada; ^5^ Department of Medicine, Faculty of Medicine, Université de Montréal, Montréal, QC, Canada

**Keywords:** tertiary lymphoid structure, antibodies, inflammation, apoptosis, injury

## Abstract

Tertiary lymphoid structures are clusters of lymphoid tissue that develop post-natally at sites of chronic inflammation. They have been described in association with infection, autoimmune disorders, cancer, and allograft rejection. In their mature stage, TLS function as ectopic germinal centers, favoring the local production of autoantibodies and cytokines. TLS formation tends to parallel the severity of tissue injury and they are usually indicative of locally active immune responses. The presence of TLS in patients with solid tumors is usually associated with a better prognosis whereas their presence predicts increased maladaptive immunologic activity in patients with autoimmune disorders or allograft transplantation. Recent data highlight a correlation between active cell death and TLS formation and maturation. Our group recently identified apoptotic exosome-like vesicles, released by apoptotic cells, as novel inducers of TLS formation. Here, we review mechanisms of TLS formation and maturation with a specific focus on the emerging importance of tissue injury, programmed cell death and extracellular vesicles in TLS biogenesis.

## Introduction

**Figure 1:**
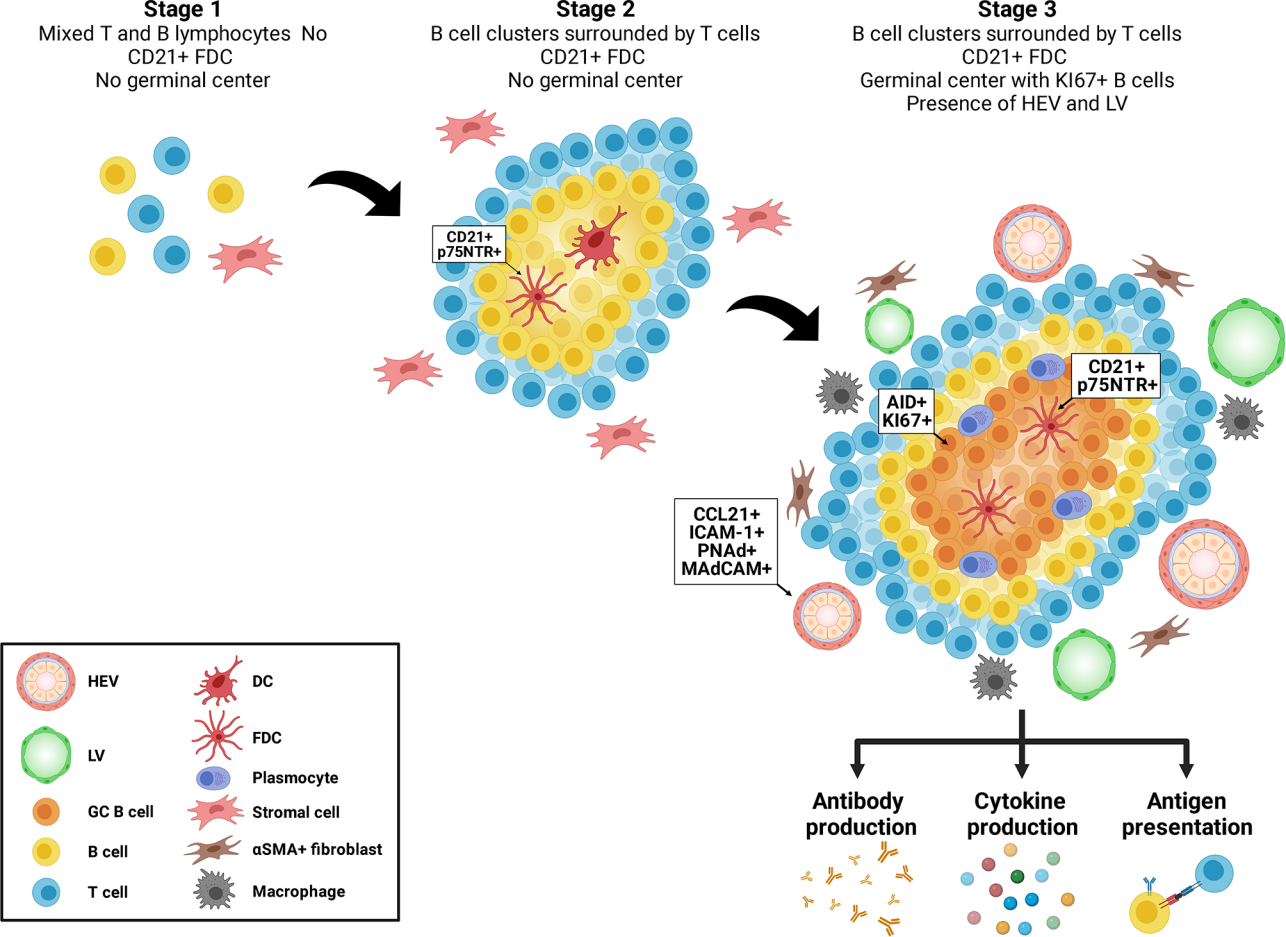
Maturation of tertiary lymphoid structures. Tertiary lymphoid structures evolve from loose aggregates of T and B cells and stromal cells (Stage 1), to polarized clusters of T and B cells accompanied by follicular dendritic cells (FDC) (Stage 2) to mature polarized structures containing germinal centers, proliferating B cells, plasma cells, high endothelial venules (HEV) and lymphoid vessels (LV).

Tertiary lymphoid structures (TLS) are ectopic aggregates of lymphocytes and stromal cells, which, at maturity, behave as functional sites of adaptive immune responses (1, 2). In contrast to secondary lymphoid organs (SLO) (such as spleen, lymph nodes and Peyer’s patches), TLS are non-encapsulated and form postnatally. They exhibit plasticity and their presence is transient, correlating with active tissue injury and resolving after antigenic clearance and tissue repair (3). They are composed of T and B cells as well as stromal cells, such as follicular dendritic cells (FDCs) and αSMA+ fibroblasts. Macrophages can be found at the periphery of TLS (4) (**Figure 1**). TLS display different organization levels ranging from simple clusters of B and T lymphocytes to more mature structures where T and B cells are polarized and FDC expressing CD21 and p75 neurotrophin receptor are present, allowing the formation of germinal centers (GC) (1, 5–7). GC are characterized by expression of activation-induced cytidine deaminase (AID) that regulated immunoglobulin gene affinity maturation through somatic hypermutation and initiation of immunoglobulin class switch recombination. GC are sites of B cell proliferation and affinity maturation into antibody secreting plasma cells. Lymphatic vessels and high endothelial venules (HEV), characterized by a cuboidal shape of endothelial cells and expression of CCL21, ICAM-1, PNAd and MAdCAM, are commonly found in mature stages (6) (**Figure 1**).

TLS arise in tissues whose main function is other than the generation of immune cells such as kidney, heart, pancreas, lung, colon and breast. Lymphoid neogenesis (5, 8), i.e. the process of TLS formation, can be observed in inflammatory microenvironments resulting from chronic infection, autoimmune conditions, allograft rejection and tumor growth (9, 10). Inflammatory cytokines such as TNF-α, IL-17A, IL-23 and lymphotoxins, expressed by immune cells at sites of injury induce stromal cells to produce homeostatic chemokines, such as CXCL13, CXCL12, CCL19 and CCL21. This in turn drives the recruitment of T and B cells and their organization into progressively polarized clusters (11). CXCL13 expression in TLS by CD8+ T cells and other immune cells appears pivotal to TLS maturation (12–16). Inflammation also prompts the expression of a number of chemokines and cytokines in tissue fibroblasts such as podoplanin, CCL19, IL-17, CXCL13, and adhesion molecules ICAM-1 and VCAM-1, therefore creating a microenvironment conducive to attraction and retention of lymphoid cells (17–22). Chemokines and cytokines produced by local fibroblasts and epithelial cells (19) favor the recruitment of immune cells and TLS organization. Various cytokines can also synergize and/or compensate one another, creating an environment favorable for TLS formation and maturation (2).

An important phase in TLS maturation is the formation of HEV that connect TLS with the bloodstream and enable the sustained recruitment of lymphocytes. HEVs express addressin and CCL21 allowing the entry of naïve T cells expressing the addressin ligand CD62L and CCR7, the chemokine receptor for CCL21 and CCL19. Data from tumor models also demonstrate that lymphotoxin α (LTα) and TNF receptor (TNFR) interactions, likely through infiltrating CD8+ T cells and NK cells, are also important for HEV formation (23). Others found that HEV formation can occur independently of both LTα and lymphotoxin (LT)-ß receptor (LTbR) (24). Specific requirements for HEV formation and TLS maturation may be a consequence of the different microenvironments in which TLS are formed. The presence of FDCs within B cell follicles is another hallmark of TLS maturation. In SLO, LTbR and TNFR signaling are essential for FDC formation. In TLS, LTα1ß2 is important for FDC generation, enabling GC formation and antigen presentation (25–27). Although FDCs progenitors remain unknown, activated local stromal cells can differentiate into FDCs upon encounter with migrating immune cells in TLSs (28).

Antigenic stimulation plays an important role in the formation of TLS and, in turn, TLS are sites of antibody formation. In numerous autoimmune diseases and alloimmune conditions, pathogenic or diagnostic autoantibodies have been shown to be produced by TLS (25, 29, 30). TLS within inflamed synovium or salivary glands in patients with rheumatoid arthritis or Sjögren’s syndrome, control the production of anti-citrullinated peptide antibody, anti-Ro/SSA and anti-La/SSB antibodies (3, 31, 32). In kidney and heart allografts with chronic rejection, TLS have been identified as a source of anti-HLA antibodies, the latter playing a major role in allograft rejection (33). Our group also recently identified a role for TLS in the production of autoantibodies that contribute to allograft inflammation and dysfunction (34, 35).

B cells within TLS can differentiate into antibody-producing plasma cells. They can also favor autoimmunity and alloimmunity by acting as antigen presenting cells, further perpetuating antigenic stimulation and immunogenicity (25, 29, 30). Some conflicting reports have pointed to the absence of correlation between TLS formation and autoimmunity or alloimmune disease activity. These results may stem from activation of tolerogenic pathways in certain TLS that harbor regulatory B and T cells (36, 37). While the presence of TLS is generally associated with disease severity in patients with autoimmunity and alloimmune diseases such as rheumatoid arthritis, Sjögren’s syndrome, IgA nephropathy and allograft rejection (31–33, 38), TLS formation in solid tumors has been generally associated with a better prognosis. B cell aggregates in tumor TLS can participate in anti-tumor immunity by serving as antigen presenting cells and by differentiating into plasma cells producing tumor-associated antibodies. TLS B cell aggregates have generally been associated with better prognosis in lung, pancreas, colon and breast cancer (39–49).

## Formation and Maturation of TLS; From Lymphotoxins to IL-17

The formation and development of SLO and TLS both rely on the expression of lymphotoxins and inflammatory cytokines such as TNFα. Lymphotoxins are members of the TNF superfamily and are pivotal to the formation of SLO. Lymphoid inducer cells (LTi) arise from innate lymphoid progenitors in the fetal liver under the tight regulation of the nuclear hormone receptor retinoic acid related orphan receptor γτ (RORγτ) and the transcriptional regulator Id2 (50, 51). LTi express lymphotoxin α2ß1 on their surface and the soluble lymphotoxin α3 form. Interactions between lymphotoxins and the LTbR on stromal cells stimulate the expression of CXCL13 and CCL21, which in turn favor homing of T and B cells. Lymphotoxins-LTbR interactions are essential for the formation and maturation of SLO as HEV and FDC require persistent LTbR mediated signaling (52, 53). LTbR stimulation was originally considered also crucial for TLSs neogenesis since LTbR expression is readily upregulated in inflamed tissues and downstream signaling directly induces lymphoid neogenesis in different models (7, 17, 20, 21, 54, 55). Further studies have shown that initial recruitment of T and B cells can occur independently of LTbR signaling (18, 56) and point to IL-17 as an important regulator of TLS biogenesis.

IL-17A is the initial member of the IL-17 cytokine family that includes IL-17A, B, C, D, E and F. The IL-17 family plays important roles in host-defense against infection and behaves as a master regulator of inflammatory and autoimmune responses. It is also known to regulate the growth of several tumors, including skin, colon, pancreas, liver, lung and myeloma (57–65). A number of immune cells can produce IL-17A including LTi, Th17 T cells and γδ T cells, which has been implicated in autoimmune and inflammatory diseases such as multiple sclerosis, psoriasis, rheumatoid arthritis, crescentic glomerulonephritis, lupus nephritis and uveitis, among others (66–82).

In multiple sclerosis, IL-17-producing γδ T cells are thought to be initiators of inflammation and inductors of Th17 cells. In the experimental autoimmune encephalomyelitis (EAE) model, early accumulation of γδ T cells was observed in the central nervous system (CNS) where they release IL-17 and IL-21 to enhance the pro-inflammatory activity of αβ Th17 cells (71). Patients with multiple sclerosis also show accumulation of IL-17+ cells in chronic demyelinated areas of the CNS, and an increase in IL-17-producing γδ T cells in the cerebrospinal fluid (72, 73). Experimental models of skin inflammation identified IL-17A/F-producing γδ T cells as necessary and sufficient to trigger psoriasis-like plaque formation in IL-23- or Immiquimod-induced models (74). IL-17-secreting γδ T cells were also shown to enhance Th17 responses when skin inflammation was triggered with BCG immunization or Freund’s adjuvant (75, 76). Similarly, human dermal γδ T cells are abundant in biopsies of psoriasis lesions, with an ability to produce higher levels of IL-17 compared to αβ Th17 cells upon IL-23 stimulation *in vitro* (74). In mouse models of non-autoimmune arthritis, resident and peripheral γδ T cells were reported as a major source of IL-17 (77, 78). An increase in circulating IL-17A-producing γδ T cells was also found in arthritis patients, suggesting their priming by cytokines secreted at the site of inflammation (79, 80). In Crescentic glomerulonephritis, renal IL-17A-producing γδ T cells were found to be the main contributor in the early inflammatory response by promoting kidney injury. They were predominated by IL-17A-producing Th17 at later phases (81). In the experimental autoimmune uveitis model, αβ and γδ T cells interactions was found to be important for mediation of eye inflammation. In this model, an early expansion of γδ T cells in SLO induces IL-17 production and further generation of Th17 responses by αβ cells at the inflammatory site (82).

A growing body of evidence has confirmed a role for IL-17A produced by Th17 T cells and γδ T cells in the development of TLS in the context of pulmonary infection, CNS inflammation, renal ischemia-reperfusion, obstruction and IgA nephropathy, and kidney transplantation (22, 38, 54, 83–88). In a model of LPS-induced pulmonary infection in neonatal mice, αβ and γδ T cells were detected within Inducible Bronchus-Associated Lymphoid Tissues (iBALT). γδ T cells formed a large proportion of infiltrating cells and both contributed to IL-17 production. Adoptive transfer of these purified T cell subsets, separately or together, to LPS-treated *Tcrbd* ^-/-^ neonatal mice, showed preferential contribution of γδ T cells in promoting iBALT development and of αβ T cells in forming larger areas of iBALT (83). Using another model of pulmonary infection induced by *Pseudomonas aeruginosa*, γδ T cells were found to be the main source of IL-17 within iBALT, inducing CXCL-12 production by IL-17R+ stromal cells, B cell recruitment and follicles formation independent of FDC. When induced in *IL-17a/f ^-/-^* or γδ T cells-deficient mice upon infection, lymphoid structures were less organized and, in the absence of γδ T cells, showed a reduction in number and size (84). In the EAE model, TLS formation in the CNS was also shown to require IL‐17 production. Among various Th cell subsets adoptively transferred to mice, IL-17-secreting podoplanin-positive Th17 cells generated large organized and well structured ectopic lymphoid follicles in the CNS (22). Renal TLOs induced by ischemia-reperfusion injury in aged mice were reported to be enriched in Th17 cell differentiation, with increased expression of IL-17A and IL-23R (38). Moreover, human renal rejected graft samples show a correlation between shorter graft survival and high interstitial infiltration of Th17 cells, producing IL-17 and IL-21 and promoting lymphoid neogenesis (85).

We have recently shown that γδ T17 cells play a critical role in IL‐17 overexpression and lymphoid neogenesis in a model of vascular rejection (34). The importance of IL‐17 in the activation of autoimmune responses in the context of transplantation appears to stem from its capacity to initiate recruitment of immune cells to sites of injury and promote maturation of antigen‐presenting cells (89–94). As Th17 cells are the classic producers of IL‐17, they have been suggested to play a pivotal role in autoimmune pathways triggered following transplantation. Intriguingly, our findings demonstrate the importance of γδ T cells, rather than Th17 cells, in coordinating the IL‐17 response triggered by vascular injury of vascular allografts (34). These observations are in line with previous studies showing that human IL‐17‐producing γδ T cells are generated in the periphery and recruited to inflamed tissues (95, 96). This process takes place more rapidly compared to the activation of conventional T lymphocytes as γδ T cells can be activated in the absence of a cognate TCR ligand (97).

Collectively, depending on the nature of the insult and the tissue implicated, peripheral or resident IL-17-producing γδ T cells may be involved at early phases to organize immunological events in response to inflammatory signals, and promote further conventional T cell responses at the site of inflammation.

## Tissue Injury, Cell Death, and Extracellular Vesicles Regulate TLS Biogenesis

The production of danger associated molecular patterns (DAMPs) at sites of injury is considered pivotal to TLS biogenesis. Various animal models and disease states in humans highlight a clear correlation between the degree of tissue injury, TLS number and maturation stages (4, 38, 98). In models of renal ischemia-reperfusion injury and ureteral obstruction in mice, the severity of renal damage is associated with TLS biogenesis. Aged mice, which show enhanced tissue injury after ischemia-reperfusion, were recently found to exhibit an increased propensity to TLS formation, translating into accentuated renal dysfunction (4, 98). Yet the precise DAMPs and mediators that are prompting TLS formation through activation of Th17 T cells and/or γδ T cells are only beginning to be characterized.

**Figure 2:**
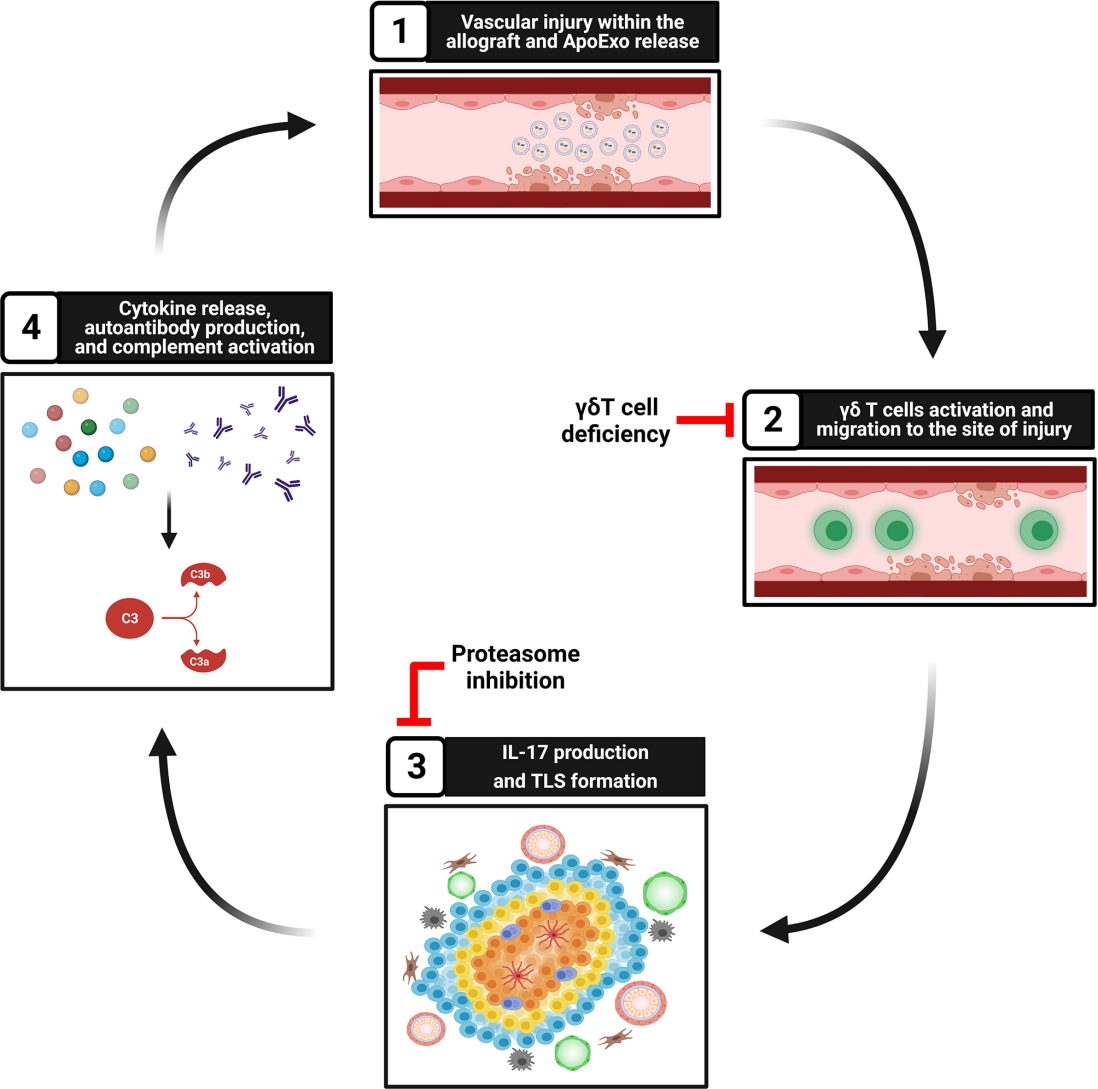
Tissue injury, ApoExo release, and TLS formation. Step 1: Vascular injury and apoptosis of endothelial cells foster the release of apoptotic exosome-like vesicles (ApoExo) carrying active 20S proteasome. Step 2: ApoExo activate γδ T cells that migrate to the site of injury. Genetic deficiency of γδ T cells decreases TLS formation and autoantibody production. Step 3: γδ T cells produce IL-17 and favor the formation of tertiary lymphoid structures at sites of injury. Inhibition of proteasome activity within ApoExo blocks TLS biogenesis and autoantibody formation. Step 4: Tertiary lymphoid structures produce proinflammatory cytokines and autoantibodies, therefore favoring complement activation and further vascular injury.

Our group and others showed that apoptosis, a type of programmed cell death classically considered non-inflammatory, can prompt the release of a number of mediators of importance in regulating immune cells towards either anti- but also pro-inflammatory and immunogenic responses (99–101). Activation of caspase-3 in dying cells leads to the release of different types of extracellular vesicles. Our group identified apoptotic exosome-like vesicles (ApoExo) as a novel type of extracellular vesicles released by endothelial cells through caspase-3 dependent pathways. ApoExo are smaller than classical apoptotic bodies, ranging from 30 to 100nm. Their protein, mRNA and microRNA contents differ from those of classical apoptotic bodies and classical exosomes (100, 102, 103). They are characterized by the presence of active 20S proteasome, perlecan LG3 C-terminal fragment and long non-coding RNAs. We showed that ApoExo are released in the bloodstream after hindlimb and renal ischemic injury resulting in higher circulating levels. In a model of vascular rejection in mice, allograft recipients injected with ApoExo showed increased TLS formation within the allograft (**Figure 2**). ApoExo injection prompted egress of γδ T cells from the spleen to the allograft leading to increased intragraft IL-17 expression, complement deposition and enhanced production of autoantibodies (34) (**Figure 2**). Mice genetically deficient in γδ T cells showed significantly less TLS formation, decreased autoantibody production and diminished allograft inflammation (**Figure 2**). Contrary to ApoExo, injection of apoptotic bodies did not foster TLS formation nor autoantibody production. The mechanism by which ApoExo activate γδ T cells and favor their homing to sites of injury remains to be fully characterized. Our results identify the proteasome activity of ApoExo as a pivotal signal regulating trafficking of γδ T cells to sites of vascular injury (34). Injection of ApoExo devoid of proteasome activity failed to induce TLS biogenesis and autoantibody formation in this system (**Figure 2**). Collectively, these recent findings identify ApoExo as novel inducers of γδ T cells activation and TLS formation and provide new clues into the mechanisms of cross talk between tissue injury and TLS biogenesis. The scope of future investigations will be to identify whether activation of γδ T cells by ApoExo is antigen specific or derives from innate signaling triggered by Toll‐like receptor ligands or nonprotein mediators.

## Conclusion

TLS are increasingly attracting interest because of their capacity to sustain local adaptive immune responses in a variety of disease states. Not only do TLS correlate with the severity and chronicity of tissue injury, they are increasingly recognized as pivotal players in maladaptive tissue remodeling, autoimmunity and inflammation. Although anti-tumor immune responses triggered and propagated from TLS are important pathways for controlling tumor growth, TLS are often associated with maladaptive autoimmune reactivity and tissue destruction in an array of autoimmune, alloimmune and chronic inflammatory diseases. The identification of ApoExo released by dying apoptotic cells as novel inducers of TLS biogenesis provides new insights into the mechanisms of cross talk that contribute to TLS formation at sites of injury.

## Author Contributions

MD, IK, and M-JH wrote the manuscript. All authors contributed to the article and approved the submitted version.

## Conflict of Interest

The authors declare that the research was conducted in the absence of any commercial or financial relationships that could be construed as a potential conflict of interest.

## References

[1] Manzo A Paoletti S Carulli M Blades MC Barone F Yanni G. Systematic Microanatomical Analysis of CXCL13 and CCL21 *In situ* Production and Progressive Lymphoid Organization in Rheumatoid Synovitis. Eur J Immunol (2005) 35(5):1347–59. 10.1002/eji.200425830 15832291

[2] Gago da Graca C van Baarsen LGM Mebius RE. Tertiary Lymphoid Structures: Diversity in Their Development, Composition, and Role. J Immunol (2021) 206(2):273–81. 10.4049/jimmunol.2000873 33397741

[3] Corsiero E Delvecchio FR Bombardieri M Pitzalis C. B Cells in the Formation of Tertiary Lymphoid Organs in Autoimmunity, Transplantation and Tumorigenesis. Curr Opin Immunol (2019) 57:46–52. 10.1016/j.coi.2019.01.004 30798069

[4] Sato Y Boor P Fukuma S Klinkhammer BM Haga H Ogawa O. Developmental Stages of Tertiary Lymphoid Tissue Reflect Local Injury and Inflammation in Mouse and Human Kidneys. Kidney Int (2020) 98(2):448–63. 10.1016/j.kint.2020.02.023 32473779

[5] Aloisi F Pujol-Borrell R. Lymphoid Neogenesis in Chronic Inflammatory Diseases. Nat Rev Immunol (2006) 6(3):205–17. 10.1038/nri1786 16498451

[6] Ruddle NH. High Endothelial Venules and Lymphatic Vessels in Tertiary Lymphoid Organs: Characteristics, Functions, and Regulation. Front Immunol (2016) 7:491. 10.3389/fimmu.2016.00491 27881983 PMC5101196

[7] Grabner R Lotzer K Dopping S Hildner M Radke D Beer M. Lymphotoxin Beta Receptor Signaling Promotes Tertiary Lymphoid Organogenesis in the Aorta Adventitia of Aged ApoE-/- Mice. J Exp Med (2009) 206(1):233–48. 10.1084/jem.20080752 PMC2626665 19139167

[8] Hjelmstrom P. Lymphoid Neogenesis: *De Novo* Formation of Lymphoid Tissue in Chronic Inflammation Through Expression of Homing Chemokines. J Leukoc Biol (2001) 69(3):331–9. 10.1189/jlb.69.3.331 11261778

[9] Pitzalis C Jones GW Bombardieri M Jones SA. Ectopic Lymphoid-Like Structures in Infection, Cancer and Autoimmunity. Nat Rev Immunol (2014) 14(7):447–62. 10.1038/nri3700 24948366

[10] Sautes-Fridman C Petitprez F Calderaro J Fridman WH. Tertiary Lymphoid Structures in the Era of Cancer Immunotherapy. Nat Rev Cancer (2019) 19(6):307–25. 10.1038/s41568-019-0144-6 31092904

[11] McDonald KG McDonough JS Dieckgraefe BK Newberry RD. Dendritic Cells Produce CXCL13 and Participate in the Development of Murine Small Intestine Lymphoid Tissues. Am J Pathol (2010) 176(5):2367–77. 10.2353/ajpath.2010.090723 PMC2861101 20304952

[12] Koscso B Kurapati S Rodrigues RR Nedjic J Gowda K Shin C. Gut-Resident CX3CR1(hi) Macrophages Induce Tertiary Lymphoid Structures and IgA Response in Situ. Sci Immunol (2020) 5(46). 10.1126/sciimmunol.aax0062 PMC7296464 32276965

[13] Thommen DS Koelzer VH Herzig P Roller A Trefny M Dimeloe S. A Transcriptionally and Functionally Distinct PD-1(+) CD8(+) T Cell Pool With Predictive Potential in non-Small-Cell Lung Cancer Treated With PD-1 Blockade. Nat Med (2018) 24(7):994–1004. 10.1038/s41591-018-0057-z 29892065 PMC6110381

[14] Manzo A Vitolo B Humby F Caporali R Jarrossay D Dell’accio F. Mature Antigen-Experienced T Helper Cells Synthesize and Secrete the B Cell Chemoattractant CXCL13 in the Inflammatory Environment of the Rheumatoid Joint. Arthritis Rheum (2008) 58(11):3377–87. 10.1002/art.23966 18975336

[15] Bellamri N Viel R Morzadec C Lecureur V Joannes A de Latour B. TNF-Alpha and IL-10 Control CXCL13 Expression in Human Macrophages. J Immunol (2020) 204(9):2492–502. 10.4049/jimmunol.1900790 32213567

[16] Carlsen HS Baekkevold ES Morton HC Haraldsen G Brandtzaeg P. Monocyte-Like and Mature Macrophages Produce CXCL13 (B Cell-Attracting Chemokine 1) in Inflammatory Lesions With Lymphoid Neogenesis. Blood (2004) 104(10):3021–7. 10.1182/blood-2004-02-0701 15284119

[17] Peduto L Dulauroy S Lochner M Spath GF Morales MA Cumano A. Inflammation Recapitulates the Ontogeny of Lymphoid Stromal Cells. J Immunol (2009) 182(9):5789–99. 10.4049/jimmunol.0803974 19380827

[18] Pikor NB Astarita JL Summers-Deluca L Galicia G Qu J Ward LA. Integration of Th17- and Lymphotoxin-Derived Signals Initiates Meningeal-Resident Stromal Cell Remodeling to Propagate Neuroinflammation. Immunity (2015) 43(6):1160–73. 10.1016/j.immuni.2015.11.010 26682987

[19] Barone F Nayar S Campos J Cloake T Withers DR Toellner KM. IL-22 Regulates Lymphoid Chemokine Production and Assembly of Tertiary Lymphoid Organs. Proc Natl Acad Sci USA (2015) 112(35):11024–9. 10.1073/pnas.1503315112 PMC4568258 26286991

[20] Link A Hardie DL Favre S Britschgi MR Adams DH Sixt M. Association of T-Zone Reticular Networks and Conduits With Ectopic Lymphoid Tissues in Mice and Humans. Am J Pathol (2011) 178(4):1662–75. 10.1016/j.ajpath.2010.12.039 PMC3070229 21435450

[21] Olivier BJ Cailotto C van der Vliet J Knippenberg M Greuter MJ Hilbers FW. Vagal Innervation is Required for the Formation of Tertiary Lymphoid Tissue in Colitis. Eur J Immunol (2016) 46(10):2467–80. 10.1002/eji.201646370 27457277

[22] Peters A Pitcher LA Sullivan JM Mitsdoerffer M Acton SE Franz B. Th17 Cells Induce Ectopic Lymphoid Follicles in Central Nervous System Tissue Inflammation. Immunity (2011) 35(6):986–96. 10.1016/j.immuni.2011.10.015 PMC3422678 22177922

[23] Peske JD Thompson ED Gemta L Baylis RA Fu YX Engelhard VH. Effector Lymphocyte-Induced Lymph Node-Like Vasculature Enables Naive T-Cell Entry Into Tumours and Enhanced Anti-Tumour Immunity. Nat Commun (2015) 6:7114. 10.1038/ncomms8114 25968334 PMC4435831

[24] Weinstein AM Chen L Brzana EA Patil PR Taylor JL Fabian KL. Tbet and IL-36gamma Cooperate in Therapeutic DC-Mediated Promotion of Ectopic Lymphoid Organogenesis in the Tumor Microenvironment. Oncoimmunology (2017) 6(6):e1322238. 10.1080/2162402X.2017.1322238 28680760 PMC5486180

[25] Hughes CE Benson RA Bedaj M Maffia P. Antigen-Presenting Cells and Antigen Presentation in Tertiary Lymphoid Organs. Front Immunol (2016) 7:481. 10.3389/fimmu.2016.00481 27872626 PMC5097899

[26] Fu YX Huang G Wang Y Chaplin DD. B Lymphocytes Induce the Formation of Follicular Dendritic Cell Clusters in a Lymphotoxin Alpha-Dependent Fashion. J Exp Med (1998) 187(7):1009–18. 10.1084/jem.187.7.1009 PMC2212211 9529317

[27] Le Pottier L Devauchelle V Fautrel A Daridon C Saraux A Youinou P. Ectopic Germinal Centers are Rare in Sjogren’s Syndrome Salivary Glands and do Not Exclude Autoreactive B Cells. J Immunol (2009) 182(6):3540–7. 10.4049/jimmunol.0803588 19265132

[28] Cupedo T Jansen W Kraal G Mebius RE. Induction of Secondary and Tertiary Lymphoid Structures in the Skin. Immunity (2004) 21(5):655–67. 10.1016/j.immuni.2004.09.006 15539152

[29] Buckley CD Barone F Nayar S Benezech C Caamano J. Stromal Cells in Chronic Inflammation and Tertiary Lymphoid Organ Formation. Annu Rev Immunol (2015) 33:715–45. 10.1146/annurev-immunol-032713-120252 25861980

[30] Pipi E Nayar S Gardner DH Colafrancesco S Smith C Barone F. Tertiary Lymphoid Structures: Autoimmunity Goes Local. Front Immunol (2018) 9:1952. 10.3389/fimmu.2018.01952 30258435 PMC6143705

[31] Humby F Bombardieri M Manzo A Kelly S Blades MC Kirkham B. Ectopic Lymphoid Structures Support Ongoing Production of Class-Switched Autoantibodies in Rheumatoid Synovium. PloS Med (2009) 6(1):e1. 10.1371/journal.pmed.0060001 PMC2621263 19143467

[32] Amara K Steen J Murray F Morbach H Fernandez-Rodriguez BM Joshua V. Monoclonal IgG Antibodies Generated From Joint-Derived B Cells of RA Patients Have a Strong Bias Toward Citrullinated Autoantigen Recognition. J Exp Med (2013) 210(3):445–55. 10.1084/jem.20121486 PMC3600900 23440041

[33] Thaunat O Field AC Dai J Louedec L Patey N Bloch MF. Lymphoid Neogenesis in Chronic Rejection: Evidence for a Local Humoral Alloimmune Response. Proc Natl Acad Sci USA (2005) 102(41):14723–8. 10.1073/pnas.0507223102 PMC1253595 16192350

[34] Dieude M Turgeon J Karakeussian Rimbaud A Beillevaire D Qi S Patey N. Extracellular Vesicles Derived From Injured Vascular Tissue Promote the Formation of Tertiary Lymphoid Structures in Vascular Allografts. Am J Transplant (2020) 20(3):726–38. 10.1111/ajt.15707 PMC7064890 31729155

[35] Cardinal H Dieude M Hebert MJ. The Emerging Importance of Non-HLA Autoantibodies in Kidney Transplant Complications. J Am Soc Nephrol (2017) 28(2):400–6. 10.1681/ASN.2016070756 PMC5280028 27798244

[36] Nova-Lamperti E Chana P Mobillo P Runglall M Kamra Y McGregor R. Increased CD40 Ligation and Reduced BCR Signalling Leads to Higher IL-10 Production in B Cells From Tolerant Kidney Transplant Patients. Transplantation (2017) 101(3):541–7. 10.1097/TP.0000000000001341 PMC5178883 27472092

[37] Brown K Sacks SH Wong W. Tertiary Lymphoid Organs in Renal Allografts can be Associated With Donor-Specific Tolerance Rather Than Rejection. Eur J Immunol (2011) 41(1):89–96. 10.1002/eji.201040759 21182080

[38] Luo R Cheng Y Chang D Liu T Liu L Pei G. Tertiary Lymphoid Organs are Associated With the Progression of Kidney Damage and Regulated by Interleukin-17A. Theranostics (2021) 11(1):117–31. 10.7150/thno.48624 PMC7681089 33391465

[39] Castino GF Cortese N Capretti G Serio S Di Caro G Mineri R. Spatial Distribution of B Cells Predicts Prognosis in Human Pancreatic Adenocarcinoma. Oncoimmunology (2016) 5(4):e1085147. 10.1080/2162402X.2015.1085147 27141376 PMC4839336

[40] Cipponi A Mercier M Seremet T Baurain JF Theate I van den Oord J. Neogenesis of Lymphoid Structures and Antibody Responses Occur in Human Melanoma Metastases. Cancer Res (2012) 72(16):3997–4007. 10.1158/0008-5472.CAN-12-1377 22850419

[41] Di Caro G Bergomas F Grizzi F Doni A Bianchi P Malesci A. Occurrence of Tertiary Lymphoid Tissue is Associated With T-Cell Infiltration and Predicts Better Prognosis in Early-Stage Colorectal Cancers. Clin Cancer Res (2014) 20(8):2147–58. 10.1158/1078-0432.CCR-13-2590 24523438

[42] Dieu-Nosjean MC Goc J Giraldo NA Sautes-Fridman C Fridman WH. Tertiary Lymphoid Structures in Cancer and Beyond. Trends Immunol (2014) 35(11):571–80. 10.1016/j.it.2014.09.006 25443495

[43] Germain C Gnjatic S Tamzalit F Knockaert S Remark R Goc J. Presence of B Cells in Tertiary Lymphoid Structures is Associated With a Protective Immunity in Patients With Lung Cancer. Am J Respir Crit Care Med (2014) 189(7):832–44. 10.1164/rccm.201309-1611OC 24484236

[44] Ladanyi A Kiss J Mohos A Somlai B Liszkay G Gilde K. Prognostic Impact of B-Cell Density in Cutaneous Melanoma. Cancer Immunol Immunother (2011) 60(12):1729–38. 10.1007/s00262-011-1071-x PMC11028465 21779876

[45] Nzula S Going JJ Stott DI. Antigen-Driven Clonal Proliferation, Somatic Hypermutation, and Selection of B Lymphocytes Infiltrating Human Ductal Breast Carcinomas. Cancer Res (2003) 63(12):3275–80. 12810659

[46] Posch F Silina K Leibl S Mundlein A Moch H Siebenhuner A. Maturation of Tertiary Lymphoid Structures and Recurrence of Stage II and III Colorectal Cancer. Oncoimmunology (2018) 7(2):e1378844. 10.1080/2162402X.2017.1378844 29416939 PMC5798199

[47] Silina K Soltermann A Attar FM Casanova R Uckeley ZM Thut H. Germinal Centers Determine the Prognostic Relevance of Tertiary Lymphoid Structures and Are Impaired by Corticosteroids in Lung Squamous Cell Carcinoma. Cancer Res (2018) 78(5):1308–20. 10.1158/0008-5472.CAN-17-1987 29279354

[48] Zhu G Falahat R Wang K Mailloux A Artzi N Mule JJ. Tumor-Associated Tertiary Lymphoid Structures: Gene-Expression Profiling and Their Bioengineering. Front Immunol (2017) 8:767. 10.3389/fimmu.2017.00767 28713385 PMC5491937

[49] Zhu W Germain C Liu Z Sebastian Y Devi P Knockaert S. A High Density of Tertiary Lymphoid Structure B Cells in Lung Tumors is Associated With Increased CD4(+) T Cell Receptor Repertoire Clonality. Oncoimmunology (2015) 4(12):e1051922. 10.1080/2162402X.2015.1051922 26587322 PMC4635865

[50] Grogan JL Ouyang W. A Role for Th17 Cells in the Regulation of Tertiary Lymphoid Follicles. Eur J Immunol (2012) 42(9):2255–62. 10.1002/eji.201242656 22949324

[51] Eberl G Marmon S Sunshine MJ Rennert PD Choi Y Littman DR. An Essential Function for the Nuclear Receptor RORgamma(t) in the Generation of Fetal Lymphoid Tissue Inducer Cells. Nat Immunol (2004) 5(1):64–73. 10.1038/ni1022 14691482

[52] Browning JL Allaire N Ngam-Ek A Notidis E Hunt J Perrin S. Lymphotoxin-Beta Receptor Signaling is Required for the Homeostatic Control of HEV Differentiation and Function. Immunity (2005) 23(5):539–50. 10.1016/j.immuni.2005.10.002 16286021

[53] Krautler NJ Kana V Kranich J Tian Y Perera D Lemm D. Follicular Dendritic Cells Emerge From Ubiquitous Perivascular Precursors. Cell (2012) 150(1):194–206. 10.1016/j.cell.2012.05.032 22770220 PMC3704230

[54] Moyron-Quiroz JE Rangel-Moreno J Kusser K Hartson L Sprague F Goodrich S. Role of Inducible Bronchus Associated Lymphoid Tissue (iBALT) in Respiratory Immunity. Nat Med (2004) 10(9):927–34. 10.1038/nm1091 15311275

[55] Lochner M Ohnmacht C Presley L Bruhns P Si-Tahar M Sawa S. Microbiota-Induced Tertiary Lymphoid Tissues Aggravate Inflammatory Disease in the Absence of RORgamma T and LTi Cells. J Exp Med (2011) 208(1):125–34. 10.1084/jem.20100052 PMC3023125 21173107

[56] Nayar S Campos J Smith CG Iannizzotto V Gardner DH Mourcin F. Immunofibroblasts are Pivotal Drivers of Tertiary Lymphoid Structure Formation and Local Pathology. Proc Natl Acad Sci USA (2019) 116(27):13490–7. 10.1073/pnas.1905301116 PMC6613169 31213547

[57] Wu L Chen X Zhao J Martin B Zepp JA Ko JS. A Novel IL-17 Signaling Pathway Controlling Keratinocyte Proliferation and Tumorigenesis *via* the TRAF4-ERK5 Axis. J Exp Med (2015) 212(10):1571–87. 10.1084/jem.20150204 PMC4577838 26347473

[58] Wang L Yi T Zhang W Pardoll DM Yu H. IL-17 Enhances Tumor Development in Carcinogen-Induced Skin Cancer. Cancer Res (2010) 70(24):10112–20. 10.1158/0008-5472.CAN-10-0775 PMC3059780 21159633

[59] Zepp JA Zhao J Liu C Bulek K Wu L Chen X. IL-17a-Induced PLET1 Expression Contributes to Tissue Repair and Colon Tumorigenesis. J Immunol (2017) 199(11):3849–57. 10.4049/jimmunol.1601540 PMC5771493 29070673

[60] Zhang Y Zoltan M Riquelme E Xu H Sahin I Castro-Pando S. Immune Cell Production of Interleukin 17 Induces Stem Cell Features of Pancreatic Intraepithelial Neoplasia Cells. Gastroenterology (2018) 155(1):210–23 e3. 10.1053/j.gastro.2018.03.041 29604293 PMC6035075

[61] Wang K Kim MK Di Caro G Wong J Shalapour S Wan J. Interleukin-17 Receptor a Signaling in Transformed Enterocytes Promotes Early Colorectal Tumorigenesis. Immunity (2014) 41(6):1052–63. 10.1016/j.immuni.2014.11.009 PMC4272447 25526314

[62] Sun C Kono H Furuya S Hara M Hirayama K Akazawa Y. Interleukin-17a Plays a Pivotal Role in Chemically Induced Hepatocellular Carcinoma in Mice. Dig Dis Sci (2016) 61(2):474–88. 10.1007/s10620-015-3888-1 26467699

[63] Jin C Lagoudas GK Zhao C Bullman S Bhutkar A Hu B. Commensal Microbiota Promote Lung Cancer Development *via* Gammadelta T Cells. Cell (2019) 176(5):998–1013 e16. 10.1016/j.cell.2018.12.040 30712876 PMC6691977

[64] Calcinotto A Brevi A Chesi M Ferrarese R Garcia Perez L Grioni M. Microbiota-Driven Interleukin-17-Producing Cells and Eosinophils Synergize to Accelerate Multiple Myeloma Progression. Nat Commun (2018) 9(1):4832. 10.1038/s41467-018-07305-8 30510245 PMC6277390

[65] Coffelt SB Kersten K Doornebal CW Weiden J Vrijland K Hau CS. IL-17-Producing Gammadelta T Cells and Neutrophils Conspire to Promote Breast Cancer Metastasis. Nature (2015) 522(7556):345–8. 10.1038/nature14282 PMC4475637 25822788

[66] Katz Y Nadiv O Beer Y. Interleukin-17 Enhances Tumor Necrosis Factor Alpha-Induced Synthesis of Interleukins 1,6, and 8 in Skin and Synovial Fibroblasts: A Possible Role as a “Fine-Tuning Cytokine” in Inflammation Processes. Arthritis Rheum (2001) 44(9):2176–84. 10.1002/1529-0131(200109)44:9<2176::AID-ART371>3.0.CO;2-4 11592383

[67] Crispin JC Oukka M Bayliss G Cohen RA Van Beek CA Stillman IE. Expanded Double Negative T Cells in Patients With Systemic Lupus Erythematosus Produce IL-17 and Infiltrate the Kidneys. J Immunol (2008) 181(12):8761–6. 10.4049/jimmunol.181.12.8761 PMC2596652 19050297

[68] Havrdova E Belova A Goloborodko A Tisserant A Wright A Wallstroem E. Activity of Secukinumab, an Anti-IL-17A Antibody, on Brain Lesions in RRMS: Results From a Randomized, Proof-of-Concept Study. J Neurol (2016) 263(7):1287–95. 10.1007/s00415-016-8128-x 27142710

[69] Nistala K Moncrieffe H Newton KR Varsani H Hunter P Wedderburn LR. Interleukin-17-Producing T Cells are Enriched in the Joints of Children With Arthritis, But Have a Reciprocal Relationship to Regulatory T Cell Numbers. Arthritis Rheum (2008) 58(3):875–87. 10.1002/art.23291 PMC2675006 18311821

[70] Chabaud M Durand JM Buchs N Fossiez F Page G Frappart L. Human Interleukin-17: A T Cell-Derived Proinflammatory Cytokine Produced by the Rheumatoid Synovium. Arthritis Rheum (1999) 42(5):963–70. 10.1002/1529-0131(199905)42:5<963::AID-ANR15>3.0.CO;2-E 10323452

[71] Sutton CE Lalor SJ Sweeney CM Brereton CF EdC L Mills KHG. Interleukin-1 and IL-23 Induce Innate IL-17 Production From γδ T Cells, Amplifying Th17 Responses and Autoimmunity. Immunity (2009) 31:331–41. 10.1016/j.immuni.2009.08.001 19682929

[72] Schirmer L Rothhammer V Hemmer B Korn T. Enriched CD161^high^ CCR6^+^ γδ T Cells in the Cerebrospinal Fluid of Patients With Multiple Sclerosis. JAMA Neurol (2013) 70(3):345–51. 10.1001/2013.jamaneurol.409 23599932

[73] Tzartos JS Friese MA Craner MJ Palace J Newcombe J Esiri MM. Interleukin-17 Production in Central Nervous System-Infiltrating T Cells and Glial Cells is Associated With Active Disease in Multiple Sclerosis. AJP (2008) 172(1):146–55. 10.2353/ajpath.2008.070690 PMC2189615 18156204

[74] Cai Y Shen X Ding C Qi C Li K Li X. Pivotal Role of Dermal IL-17-Producing γδ T Cells in Skin Inflammation. Immunity (2011) 35:596–610. 10.1016/j.immuni.2011.08.001 21982596 PMC3205267

[75] Sumaria N Roediger B Ng LG Qin J Pinto R Cavanagh LL. Cutaneous Immunosurveillance by Self-Renewing Dermal γδ T Cells. J Exp Med (2011) 208(3):505–18. 10.1084/jem.20101824 PMC3058585 21339323

[76] Roark CL Huang Y Jin N Aydintug MK Casper T Sun D. A Canonical Vγ4vδ4+ γδ T Cell Population With Distinct Stimulation Requirements Which Promotes the Th17 Response. Immunol Res (2013) 55:217–30. 10.1007/s12026-012-8364-9 PMC3543513 22961659

[77] Reinhardt A Yevsa T Worbs T Lienenklaus S Sandrock I Oberdörfer L. Interleukin-23–Dependent γ/δ T Cells Produce Interleukin-17 and Accumulate in the Enthesis, Aortic Valve, and Ciliary Body in Mice. Arthritis Rheumatol (2016) 68(10):2476–86. 10.1002/art.39732 27111864

[78] Avau A Mitera T Put S Put K Brisse E Filtjens J. Systemic Juvenile Idiopathic Arthritis–Like Syndrome in Mice Following Stimulation of the Immune System With Freund’s Complete Adjuvant. Arthritis Rheumatol (2014) 66(5):1340–51. 10.1002/art.38359 24470407

[79] Kenna TJ Davidson SI Duan R Bradbury LA McFarlane J Smith M. Enrichment of Circulating Interleukin-17–Secreting Interleukin-23 Receptor-Positive γ/δ T Cells in Patients With Active Ankylosing Spondylitis. Arthritis Rheumatol (2012) 64(5):1420–9. 10.1002/art.33507 22144400

[80] Kessel C Lippitz K Weinhage T Hinze C Wittkowski H Holzinger D. Proinflammatory Cytokine Environments can Drive Interleukin-17 Overexpression by γ/δ T Cells in Systemic Juvenile Idiopathic Arthritis. Arthritis Rheumatol (2017) 69(7):1480–94. 10.1002/art.40099 28296284

[81] Turner J-E Krebs C Tittel AP Paust H-J Meyer-Schwesinger C Bennstein SB. IL-17A Production by Renal γδ T Cells Promotes Kidney Injury in Crescentic GN. J Am Soc Nephrol (2012) 23(9):1486–95. 10.1681/ASN.2012010040 PMC3431412 22797181

[82] Cui Y Shao H Lan C Nian H O’Brien RL Born WK. Major Role of Gd T Cells in the Generation of IL-17^+^ Uveitogenic T Cells. J Immunol (2009) 183:560–7. 10.4049/jimmunol.0900241 PMC4077214 19542467

[83] Rangel-Moreno J Carragher DM de la Luz Garcia-Hernandez M Hwang JY Kusser K Hartson L. The Development of Inducible Bronchus-Associated Lymphoid Tissue Depends on IL-17. Nat Immunol (2011) 12(7):639–46. 10.1038/ni.2053 PMC3520063 21666689

[84] Fleige H Ravens S Moschovakis GL Bölter J Willenzon S Sutter G. IL-17–Induced CXCL12 Recruits B Cells and Induces Follicle Formation in BALT in the Absence of Differentiated FDCs. J Exp Med (2014) 21(4):643–51. 10.1084/jem.20131737 PMC3978277 24663215

[85] Deteix C Attuil-Audenis V Duthey A Patey N McGregor B Dubois V. Intragraft Th17 Infiltrate Promotes Lymphoid Neogenesis and Hastens Clinical Chronic Rejection. J Immunol (2010) 184(9):5344–51. 10.4049/jimmunol.0902999 20357253

[86] Zhang X Lu B. IL-17 Initiates Tertiary Lymphoid Organ Formation. Cell Mol Immunol (2012) 9(1):9–10. 10.1038/cmi.2011.48 22179673 PMC4002935

[87] Cupedo T. An Unexpected Role for IL-17 in Lymphoid Organogenesis. Nat Immunol (2011) 12(7):590–2. 10.1038/ni.2058 21685952

[88] Patakas A Benson RA Withers DR Conigliaro P McInnes IB Brewer JM. Th17 Effector Cells Support B Cell Responses Outside of Germinal Centres. PloS One (2012) 7(11):e49715. 10.1371/journal.pone.0049715 23166752 PMC3500323

[89] Burlingham WJ Love RB Jankowska-Gan E Haynes LD Xu Q Bobadilla JL. IL-17-Dependent Cellular Immunity to Collagen Type V Predisposes to Obliterative Bronchiolitis in Human Lung Transplants. J Clin Invest (2007) 117(11):3498–506. 10.1172/JCI28031 PMC2040314 17965778

[90] Saini D Weber J Ramachandran S Phelan D Tiriveedhi V Liu M. Alloimmunity-Induced Autoimmunity as a Potential Mechanism in the Pathogenesis of Chronic Rejection of Human Lung Allografts. J Heart Lung Transplant (2011) 30(6):624–31. 10.1016/j.healun.2011.01.708 PMC3091959 21414808

[91] Goers TA Ramachandran S Aloush A Trulock E Patterson GA Mohanakumar T. De Novo Production of K-Alpha1 Tubulin-Specific Antibodies: Role in Chronic Lung Allograft Rejection. J Immunol (2008) 180(7):4487–94. 10.4049/jimmunol.180.7.4487 PMC2796833 18354170

[92] Hachem RR Tiriveedhi V Patterson GA Aloush A Trulock EP Mohanakumar T. Antibodies to K-Alpha 1 Tubulin and Collagen V are Associated With Chronic Rejection After Lung Transplantation. Am J Transplant (2012) 12(8):2164–71. 10.1111/j.1600-6143.2012.04079.x PMC3409301 22568593

[93] Fukami N Ramachandran S Saini D Walter M Chapman W Patterson GA. Antibodies to MHC Class I Induce Autoimmunity: Role in the Pathogenesis of Chronic Rejection. J Immunol (2009) 182(1):309–18. 10.4049/jimmunol.182.1.309 PMC2802821 19109162

[94] Nath DS Ilias Basha H Tiriveedhi V Alur C Phelan D Ewald GA. Characterization of Immune Responses to Cardiac Self-Antigens Myosin and Vimentin in Human Cardiac Allograft Recipients With Antibody-Mediated Rejection and Cardiac Allograft Vasculopathy. J Heart Lung Transplant (2010) 29(11):1277–85. 10.1016/j.healun.2010.05.025 PMC2963681 20615726

[95] Papotto PH Reinhardt A Prinz I Silva-Santos B. Innately Versatile: Gammadelta17 T Cells in Inflammatory and Autoimmune Diseases. J Autoimmun (2018) 87:26–37. 10.1016/j.jaut.2017.11.006 29203226

[96] Caccamo N La Mendola C Orlando V Meraviglia S Todaro M Stassi G. Differentiation, Phenotype, and Function of Interleukin-17-Producing Human Vgamma9Vdelta2 T Cells. Blood (2011) 118(1):129–38. 10.1182/blood-2011-01-331298 21505189

[97] Shiromizu CM Jancic CC. Gammadelta T Lymphocytes: An Effector Cell in Autoimmunity and Infection. Front Immunol (2018) 9:2389. 10.3389/fimmu.2018.02389 30386339 PMC6198062

[98] Sato Y Mii A Hamazaki Y Fujita H Nakata H Masuda K. Heterogeneous Fibroblasts Underlie Age-Dependent Tertiary Lymphoid Tissues in the Kidney. JCI Insight (2016) 1(11):e87680. 10.1172/jci.insight.87680 27699223 PMC5033938

[99] Sirois I Raymond MA Brassard N Cailhier JF Fedjaev M Hamelin K. Caspase-3-Dependent Export of TCTP: A Novel Pathway for Antiapoptotic Intercellular Communication. Cell Death Differ (2011) 18(3):549–62. 10.1038/cdd.2010.126 PMC3131994 20966960

[100] Dieude M Bell C Turgeon J Beillevaire D Pomerleau L Yang B. The 20S Proteasome Core, Active Within Apoptotic Exosome-Like Vesicles, Induces Autoantibody Production and Accelerates Rejection. Sci Transl Med (2015) 7(318):318ra200. 10.1126/scitranslmed.aac9816 26676607

[101] Dieude M Cardinal H Hebert MJ. Injury Derived Autoimmunity: Anti-Perlecan/LG3 Antibodies in Transplantation. Hum Immunol (2019) 80(8):608–13. 10.1016/j.humimm.2019.04.009 31029511

[102] Migneault F Dieude M Turgeon J Beillevaire D Hardy MP Brodeur A. Apoptotic Exosome-Like Vesicles Regulate Endothelial Gene Expression, Inflammatory Signaling, and Function Through the NF-kappaB Signaling Pathway. Sci Rep (2020) 10(1):12562. 10.1038/s41598-020-69548-0 32724121 PMC7387353

[103] Hardy MP Audemard E Migneault F Feghaly A Brochu S Gendron P. Apoptotic Endothelial Cells Release Small Extracellular Vesicles Loaded With Immunostimulatory Viral-Like RNAs. Sci Rep (2019) 9(1):7203. 10.1038/s41598-019-43591-y 31076589 PMC6510763

